# Plasmonic Metasurfaces Based on Pyramidal Nanoholes for High-Efficiency SERS Biosensing

**DOI:** 10.1021/acsami.1c12525

**Published:** 2021-09-01

**Authors:** Giovanna Palermo, Massimo Rippa, Ylli Conti, Ambra Vestri, Riccardo Castagna, Giovanna Fusco, Elisabetta Suffredini, Jun Zhou, Joseph Zyss, Antonio De Luca, Lucia Petti

**Affiliations:** † Department of Physics, University of Calabria, Via P. Bucci, 87036 Rende, CS, Italy; ‡ CNR NANOTEC—Istituto di Nanotecnologia, UOS Cosenza, 87036 Rende, CS, Italy; § Institute of Applied Sciences and Intelligent Systems ”E. Caianiello” CNR, 80078 Pozzuoli, Italy; ∥ Department of Food Safety, Nutrition and Veterinary Public Health, Istituto Superiore di Sanitá, 00161 Rome, Italy; ⊥ Institute of Photonics, Faculty of Science, Ningbo University, 315211 Ningbo, People’s Republic of China; # LUMIN Laboratory (CNRS), Institut d’Alembert, Universitè Paris Saclay, 91190 Gif sur Yvette, France

**Keywords:** surface-enhanced Raman scattering, metasurface, plasmonics, biosensing, pyramidal nanoholes, hepatitis A virus

## Abstract

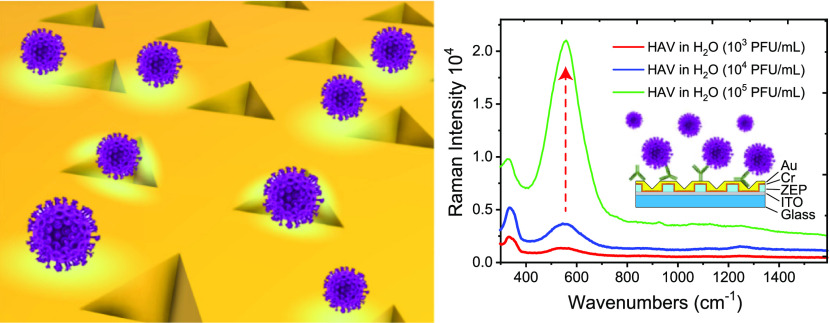

An inverted pyramidal metasurface was designed, fabricated, and studied at the nanoscale level for the development of a label-free pathogen detection on a chip platform that merges nanotechnology and surface-enhanced Raman scattering (SERS). Based on the integration and synergy of these ingredients, a virus immunoassay was proposed as a relevant proof of concept for very sensitive detection of hepatitis A virus, for the first time to our best knowledge, in a very small volume (2 μL), without complex signal amplification, allowing to detect a minimal virus concentration of 13 pg/mL. The proposed work aims to develop a high-flux and high-accuracy surface-enhanced Raman spectroscopy (SERS) nanobiosensor for the detection of pathogens to provide an effective method for early and easy water monitoring, which can be fast and convenient.

## Introduction

Detection of pathogens present in food and water is essential to help ensure food safety. In recent years, the progress of technology and applications of plasmonic nanomaterials, as well as surface-enhanced Raman scattering (SERS) effect, allowed developing an effective method to achieve high flux, high accuracy, and high sensitivity for analytical purposes and pathogen detection.^1−5^ Furthermore, the last decade has been characterized by the development of artificial materials (metamaterials—MMTs)^6−8^ and metasurfaces,^9,10^ offering an advanced and unprecedented control of the interaction between the electromagnetic (EM) field and biological matter, thereby enabling the design of increasingly innovative nanosensors.^11−17^ Plasmonic technologies represent a rapidly growing field that allow to develop valid alternatives to conventional diagnostic methods. Virus diagnosis is mainly carried out using various molecular techniques based on polymerase chain reaction (PCR), in situ hybridization process, as well as enzyme-linked immunosorbent assay (ELISA).^18^ Although these methods have many advantages and are widely used, they face some limitations. Specifically, they require multiple sampling tests and reagents, complicated multistep procedures, and highly specialized staff, entailing high costs. Moreover, the time-consuming nature and/or the need for sophisticated instrumentation of those methods limit their on-site applications. It is, therefore, necessary to identify new analytical routes, which are at the same time sensitive, specific, have short execution times, and can be used in the screening analysis of water samples and as an alternative to conventional methods. Considerable progress has been made in the past 5–10 years in the development of plasmonic nanobiosensors for detection of viruses,^19−26^ and the possibility to discriminate different viruses, and even different strains of the same virus, by SERS fingerprint has already been successfully reported in the literature.^27−29^

In this context, hepatitis A virus (HAV), belonging to the Picornaviridae family, stands out as a waterborne and foodborne human pathogen, responsible for about half of the total number of hepatitis infections diagnosed worldwide.^30−36^ To date, there are very few alternative methods stated in the literature based mainly on electrochemical and optical biosensors for HAV detection.^37,38^

Plasmonic properties of metallic nanoparticles have been used to detect viruses with different approaches;^28^ however, there seems to be a lack of studies concerning plasmonic devices for the direct detection of the whole HAV. In 2002, Cao et al. developed a system based on gold nanoparticles to detect HAV nucleic acids using a SERS spectroscopy method.^39^ In 2009, Jang et al. reported a restriction-enzyme-coded DNA detection method based on gold nanoparticle probes for the HAV DNA target.^40^ In 2014, Liu et al. developed a one-tube colorimetric platform based on LSPR-active nanoparticles sensitive to oligonucleotides related to a specific HAV gene.^41^ In 2017, Su et al. used a method based on DNA-mediated gold–silver nanoparticles for the simultaneous SERS detection of various targets, including HAV nucleic acids.^42^

Our work aims at introducing innovative engineered metasurfaces that resulted in highly sensitive, selective, and reliable pathogen detection methods. Herein, we report on the engineering of a novel plasmonic metasurface based on inversed pyramidal nanoholes (P-NHs)^43−49^ that can be used as a plasmonic substrate for sensitive SERS analysis^1−4^ and tested, as a proof of concept, for the analysis and detection of HAV in water. The considered P-NH pattern, based on a periodic geometry of nanocavities, presents some important features as compared to other patterns reported in the literature. In comparison to other patterns based on nanoholes (where no gold is present in the holes), our nanostructure consists of a real continuous metal layer with a plasmonic material present also in the cavities (FF = 1). This last property (i) maximizes the area available for functionalization, (ii) makes the plasmonic field active for sensing also inside the cavities, and (iii) allows to reduce the fluorescence signal generated by both the layers below the surface and by the glass substrate. The P-NH nanopattern that we propose is hexagonal based. Such arrangement enables a higher packing factor of the unit cell and so, combined with its smaller dimensions, a higher density of cells (and therefore of hot spots) compared to commercial substrates, such as those belonging to the Klarite family.^45−47^ It is worthwhile to underline that this difference is important in terms of hot spot intensity. In fact, the vertices of the triangular base of the proposed pyramid (characterized by an angular aperture of 60°) provide a higher-intensity hot spot than those of the square base (angular aperture 90°) of the Klarite substrates. Moreover, the lower area of our pyramids (6.59 × 10^–8^ mm^2^) than the pyramids of the Klarite substrates (2.16 × 10^–6^ mm^2^ for the standard Klarite 302 and 2.16 × 10^–6^ mm^2^ for the next-generation Klarite 308) favors virus detection, as reported in this study. We studied the geometries with different interdistances among the P-NHs by means of simulations based on a finite element method (FEM) and fabricated them with the electron beam lithography (EBL) technique. Realized nanostructures and metasurfaces were morphologically characterized using scanning electron microscopy (SEM) and atomic force microscopy (AFM) techniques and analyzed in near and far fields using scanning near-field optical microscopy (SNOM) and vis–NIR spectroscopy, respectively. As a result, we demonstrated the possibility to achieve the SERS fingerprint of the physioabsorbed HAV in water with an extremely low sample volume (2 μL). It is worthwhile to underline that it was chosen to detect HAV in water since enteric viruses are normally present in aquatic environments.^50,51^ As a proof of concept, we also functionalized our metasurface using a HAV antibody to prove that our engineered substrate was suitable for the development of a biosensor exploitable for the quantitative detection of HAV by SERS. In particular, we used the biofunctionalized metasurface to carry out SERS measurements in the presence of various HAV concentrations to detect a minimum quantity of 13 pg/mL. A different virus, Murine Norovirus (MuNoV), was finally used to preliminarily evaluate the specificity of our immunosurface. Our immuno approach allows to detect the whole virus without any need of nucleic acid extraction steps, thus making it feasible for both fast and easy detection and in situ analysis with portable systems. This is the first time, to our best knowledge, that a plasmonic engineered substrate is used for both the SERS fingerprint identification and the immuno-SERS detection of HAV.

## Results and Discussion

**Figure 1:**
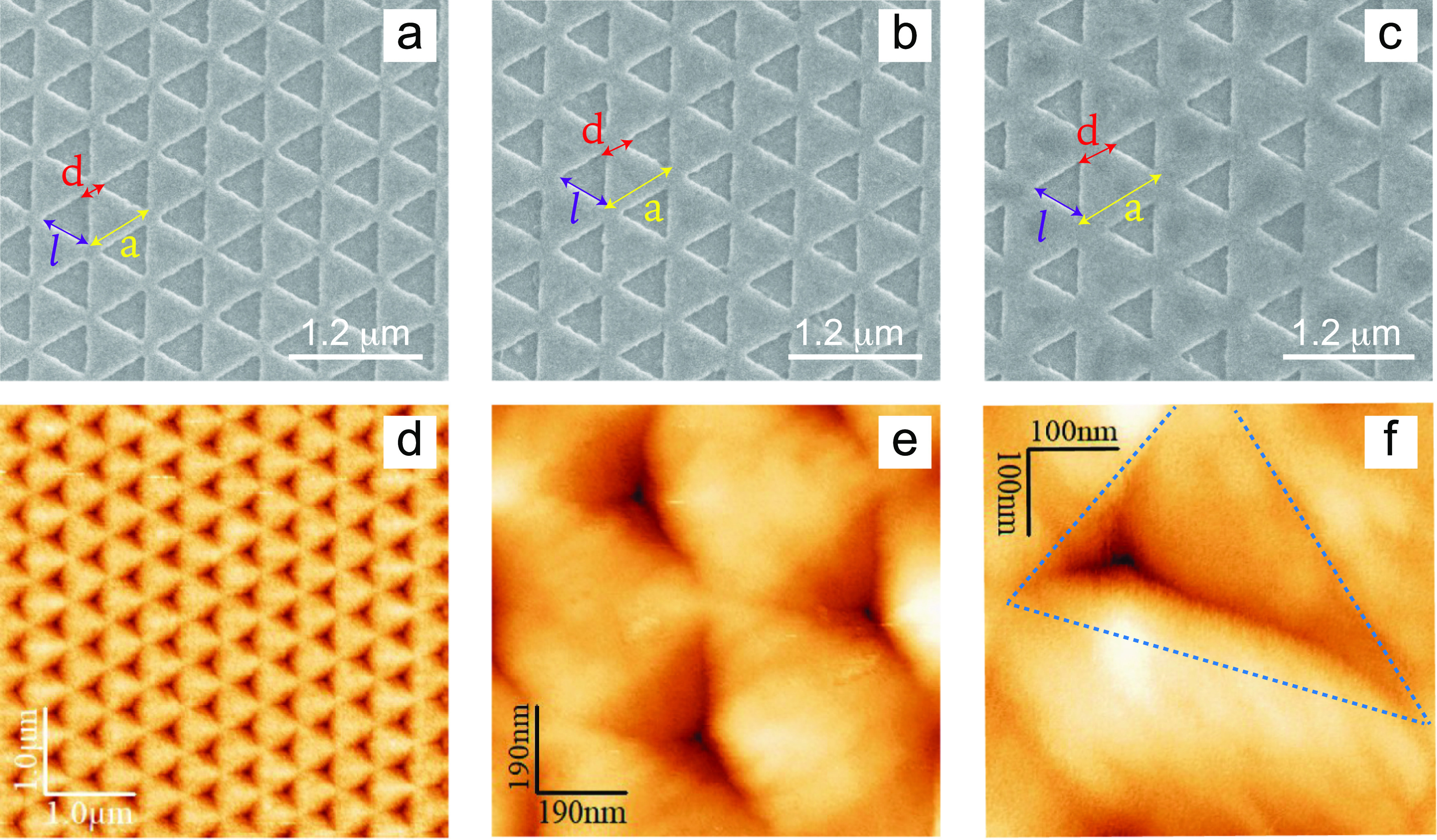
Scanning electron microscopy (SEM) images of Au nanopyramid samples: (a) A1 (*d* = 110 nm, *a* = 500 nm), (b) B1 (*d* = 160 nm, *a* = 550 nm), and (c) C1 (*d* = 250 nm, *a* = 640 nm). The side: *l*= 390 nm. White bar is 1.2 μm. Atomic force microscopy (AFM) images with different magnification of sample A1: (d) 5 × 5 μm^2^, (e) 1 × 1 μm^2^, and (f) 500 × 500 nm^2^.

Metasurfaces operating in the frequency band of the visible range have been fabricated based on gold inverted pyramidal nanoholes (P-NHs) using a high-resolution electron beam lithography (EBL) process (see the Materials and Methods section for details). The fabricated samples, characterized by a hexagonal arrangement of the P-NHs and different geometrical parameters, are identified as A1 (edge–edge interdistance *d*= 110 nm, red arrow in Figure 1a, period *a*= 500 nm, yellow arrow in Figure 1a), B1 (*d*= 160 nm, *a*= 550 nm), and C1 (*d*= 250 nm, *a*= 640 nm). The side *l* of the triangular base is fixed to 390 nm for all of the three samples.

The surface analysis of all of the fabricated samples has been carried out using a scanning electron microscope (SEM) and an atomic force microscope (AFM). The SEM analysis is reported in Figure 1a–c for the samples A1, B1, and C1, respectively. They provide evidence for the high uniformity and regularity of the triangular geometries over the whole patterned area. The resulting inverted pyramidal shape of the nanostructures is evidenced by the AFM analysis, which enables to access the wedge profile of the pyramid on its three sides. In particular, we report the AFM images with different magnifications of the sample (see Figure 1d,e). In the Supporting Information, a cut of the AFM image is reported (Figure S1), evidencing a ca. 50 nm deep inverted pyramidal shape.

The transmitted field has been acquired in the far field by a spectrometer (see the Materials and Methods section) in the visible range to study the characteristic plasmonic modes of the three metasurfaces. Extinction spectra, reported in the Supporting Information (Figure S2), show a predominant absorbance peak around 532 nm, typical of gold, whereas other two peaks around 650 and 750 nm are mainly related to the coupling between the nanocavities. By increasing the interdistance among them, a red shift of these secondary resonances occurs (see Figure S2 in the Supporting Information). To ascertain the nature of these modes, a numerical analysis with Comsol Multiphysics has been conducted. In particular, the absorbance, transmittance, and reflectance have been calculated for an Au layer taking the same value for the thickness of the metasurface and a silica metasurface displaying the same arrangement for the P-NHs and for the Au P-NHs metasurfaces, with a single P-NH in the unit cell arranged in the same hexagonal template followed throughout this study (see the Supporting Information—Figure S3).

It is well known that the main feature of surface plasmon polaritons (SPPs), that is, cavity plasmon resonances and their coupling, arises in the near-field propagating regime. In fact, scanning near-field optical microscopy (SNOM) enables the opportunity to visualize the surface plasmon-mediated mechanism, as well as the enhanced transmission phenomenon, through the particular patterned structures, beyond the diffraction limit.^52^ To this end, a SNOM analysis has been carried out on the three samples by considering the typical excitation wavelength for gold (λ_exc_ = 532 nm) at different incident polarizations. In addition to the typical *x* and *y* polarizations (not reported here), with respect to the P-NHs orientation, we considered the intermediate case of a 45° polarization orientation.

**Figure 2:**
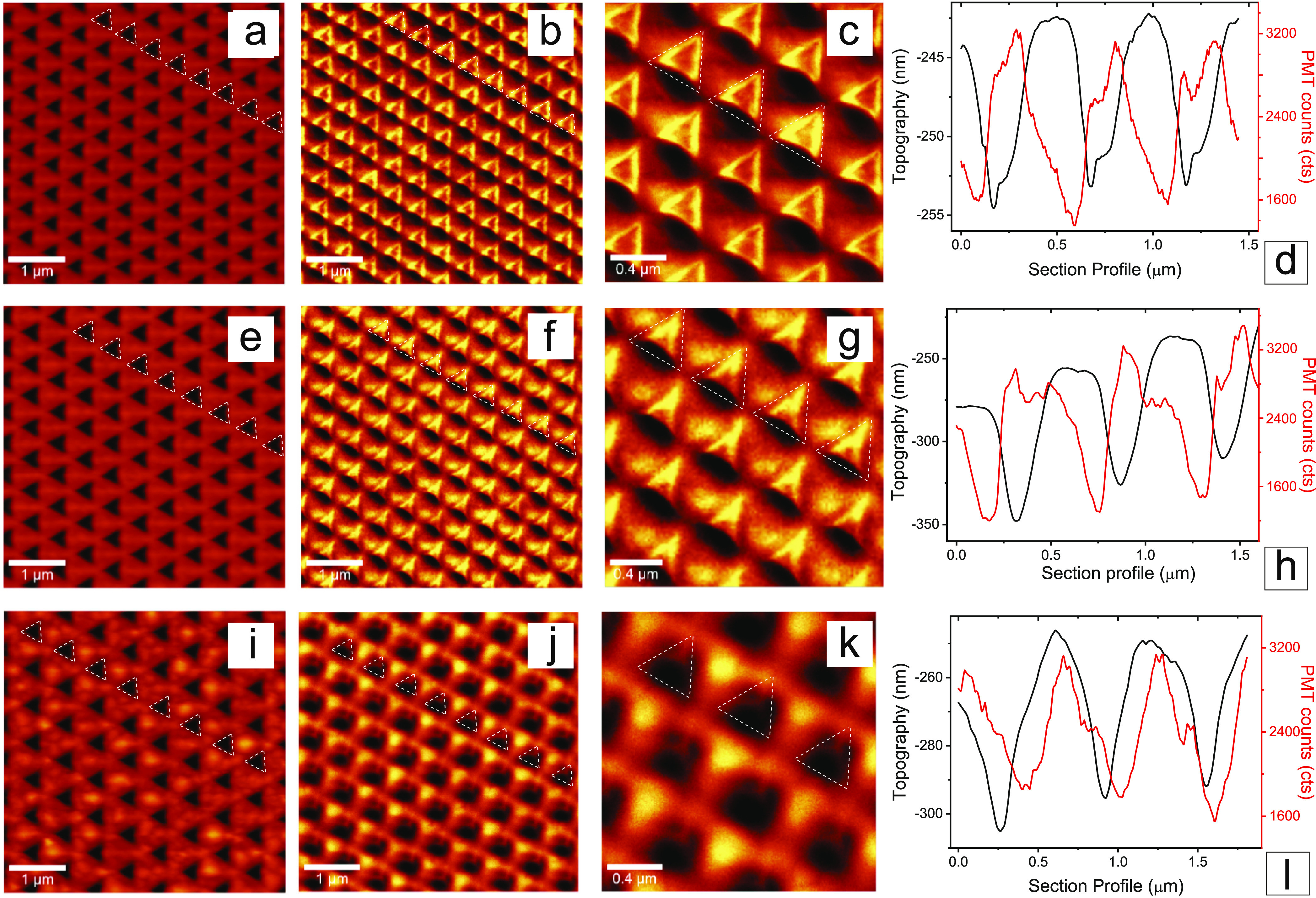
Polarization-dependent near-field response of Au nanopyramid samples. (a) Topography and (b, c) the corresponding SNOM images with (d) line profiles of topography and SNOM images along the white line from (a) and (b) of sample A1, when excited by a 45° polarized 532 nm CW laser beam. (e) Topography and (f, g) the corresponding SNOM images with (h) line profiles of topography and SNOM images along the white line from (e) and (f) of sample B1, when excited by a 45° polarized 532 nm laser beam. (i) Topography and (j, k) the corresponding SNOM images with (l) line profiles of topography and SNOM images along the white line from (i) and (j) of sample C1, when excited by a 45° polarized 532 nm laser beam.

Then, the transmitted signal through the three P-NHs has been acquired under the same experimental conditions (incident power and polarization of excitation laser source) in the SNOM–AFM combination mode. In this way, it is possible to obtain a topographic image with the corresponding SNOM analysis. Figure 2a,e,i reports the AFM topography of the three samples, obtained during the SNOM scan. The near-field optical analysis of sample A1 is reported in Figure 2b,c (Figure 2f,g for sample B1 and Figure 2j,k for sample C1). The three P-NH metasurfaces show the formation of electromagnetic hot spots in different regions of the samples. As we can see from the SNOM analysis, the interdistance among the P-NHs plays a fundamental role in the formation and localization of the hot spots on the metasurface. It is well known that the overall plasmonic modes observed on a metal film combine the surface plasmonic modes that propagate at the metal/dielectric interfaces on both sides of the excited film. In the presence of arrays of nanocavities, the SPP plasmonic modes that propagate on the surfaces can couple with the resonant modes localized in the cavity, confining part of the energy in it and intensifying the signal that is transmitted through it^52^ or in the gap^53,54^ between the nanocavities, depending on the interdistance between the nanostructures.

This behavior is shown from sample A1 (Figure 2b,c), where the modes appear to be strongly confined inside the P-NHs, in particular on the edges of the nanocavities. This sample exhibits a minimal edge distance between the P-NHs and turns out to be the most efficient one in terms of the spatial confinement of the EM field when compared to the other two samples. This is made even more evident by extracting the line profiles from the topography and SNOM images (Figure 2d), with the maximum SNOM signal (photomultiplier tube, PMT counts in red) reaching close to the edge of the topographic line profile. A different situation is detected for sample B1, characterized by values of *d* = 160 nm and *a* = 550 nm, increased by 50 nm compared to sample A1 (topography in Figure 2e). For this sample, the hot spots appear to be mainly confined inside the P-NHs, with a less intense near-field signal even in the gap regions between the nanocavities (Figure 2f–h). A third and wholly different situation arises for sample C1 (Figure 2i, characterized by *d* = 250 nm and *a* = 640, 90 nm more than sample B1): the near-field signal is then located totally out of the P-NHs and confined in the gaps in-between (Figure 2j,k). The line profiles of the topography and SNOM images (Figure 2l) confirm this behavior, with a maximum of the SNOM signal overlapping the gold gap region between the P-NHs. The SNOM analysis of the three samples reveals that a modulation of the metasurface optical response can be obtained by varying the distance between the nanocavities while keeping the dimensions of the P-NHs fixed. We can thus distinguish a first case in which the EM hot spots can be totally confined in the cavities (sample A1), a uniform distribution of the hot spots between the nanocavities and the gaps region (sample B1), and a third case in which the hot spots are totally confined in the gaps region (sample C1).

**Figure 3:**
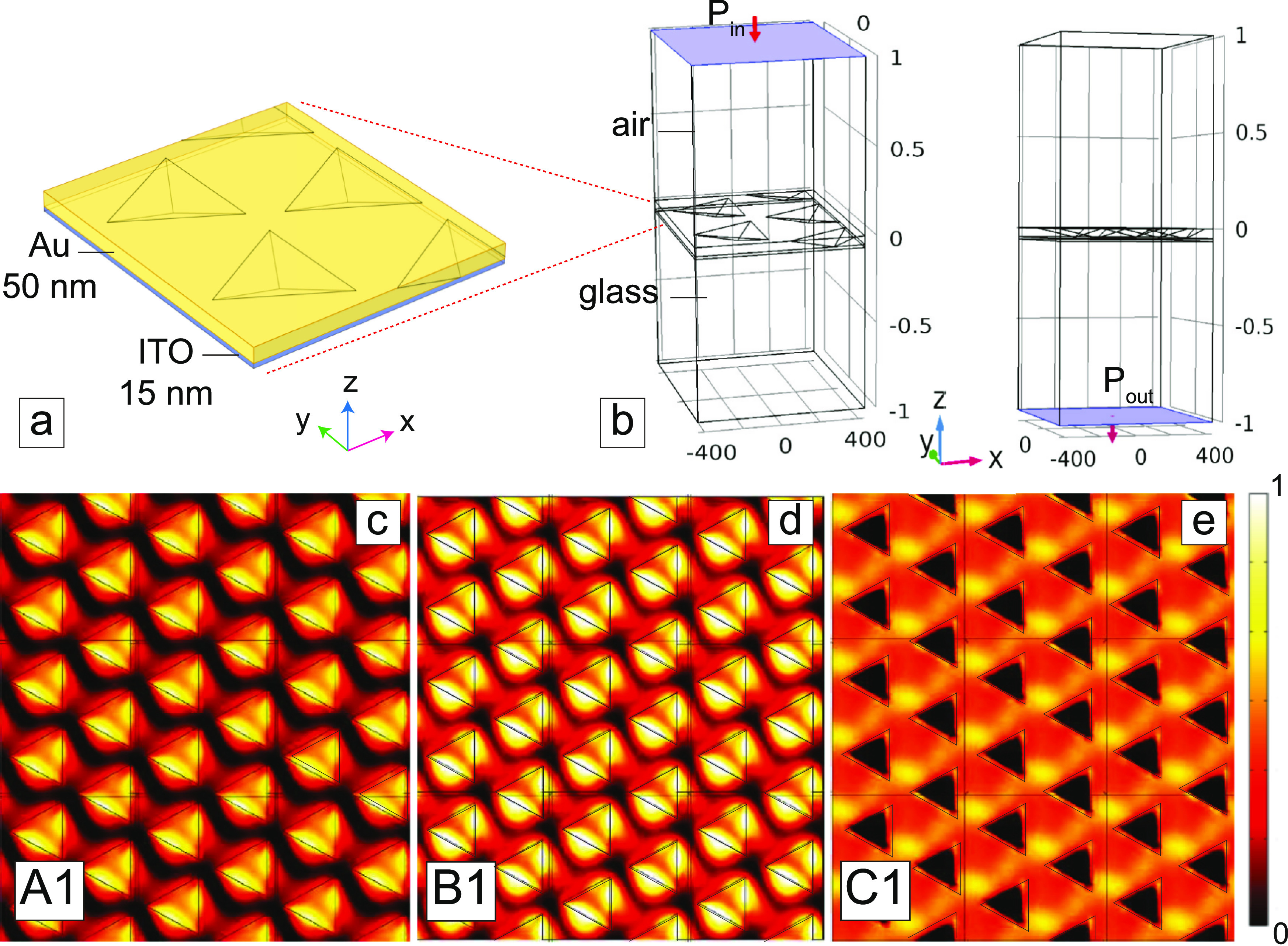
(a) Unit cell of the Au nanopyramid array simulated geometry. (b) COMSOL allows to simulate sources or detectors of EM radiation by creating ports. Simulated near-field enhancement maps (|**E**|/*E*_0_) in the metasurface volume of samples (c) A1, (d) B1, and (e) C1 for a 45° polarized 532 nm incident light beam.

To further prove the significant role of the interdistance between the P-NHs in the development of metasurfaces with controlled EM hot spots, we performed numerical simulations based on a finite element method (FEM) on three different geometries representative of the samples analyzed experimentally. At this end, a unit cell is built in COMSOL Multiphysics and reported in Figure 3a,b. The simulated domain is characterized by periodic conditions on both sides of the parallelepiped to simulate an infinite array of the unit cell.

The software permits to simulate sources or detectors of EM radiation by creating ports: in our case, a port on the top (P_in_) represents the input, from which the radiation starts to propagate, whereas a port on the bottom (*P*_out_), behaves as a detector (Figure 3b). For details related to the numerical analysis, see the “Numerical Simulations: Finite Element Method” in the Materials and Methods section. Figure 3c–e presents the 2D surface maps of the cumulative amplitude of the electric field (), normalized to the incident electric field *E*_0_ in the entire metasurface volume (with 0 < *z* < -50 nm, 0 nm corresponding to the gold top surface) for the three reported simulated geometries. As a result of the FEM analysis, it is possible to well distinguish bright modes inside each nanocavity for the simulated domain with geometrical parameters in accordance with sample A1, which moves in the edge regions and outside the nanocavities for the geometry corresponding to sample B1, while for the third geometry (corresponding to sample C1), it results in the confinement of hot spots within the nanocavities. Numerical simulations confirm the experimental results from the SNOM characterization.

**Figure 4:**
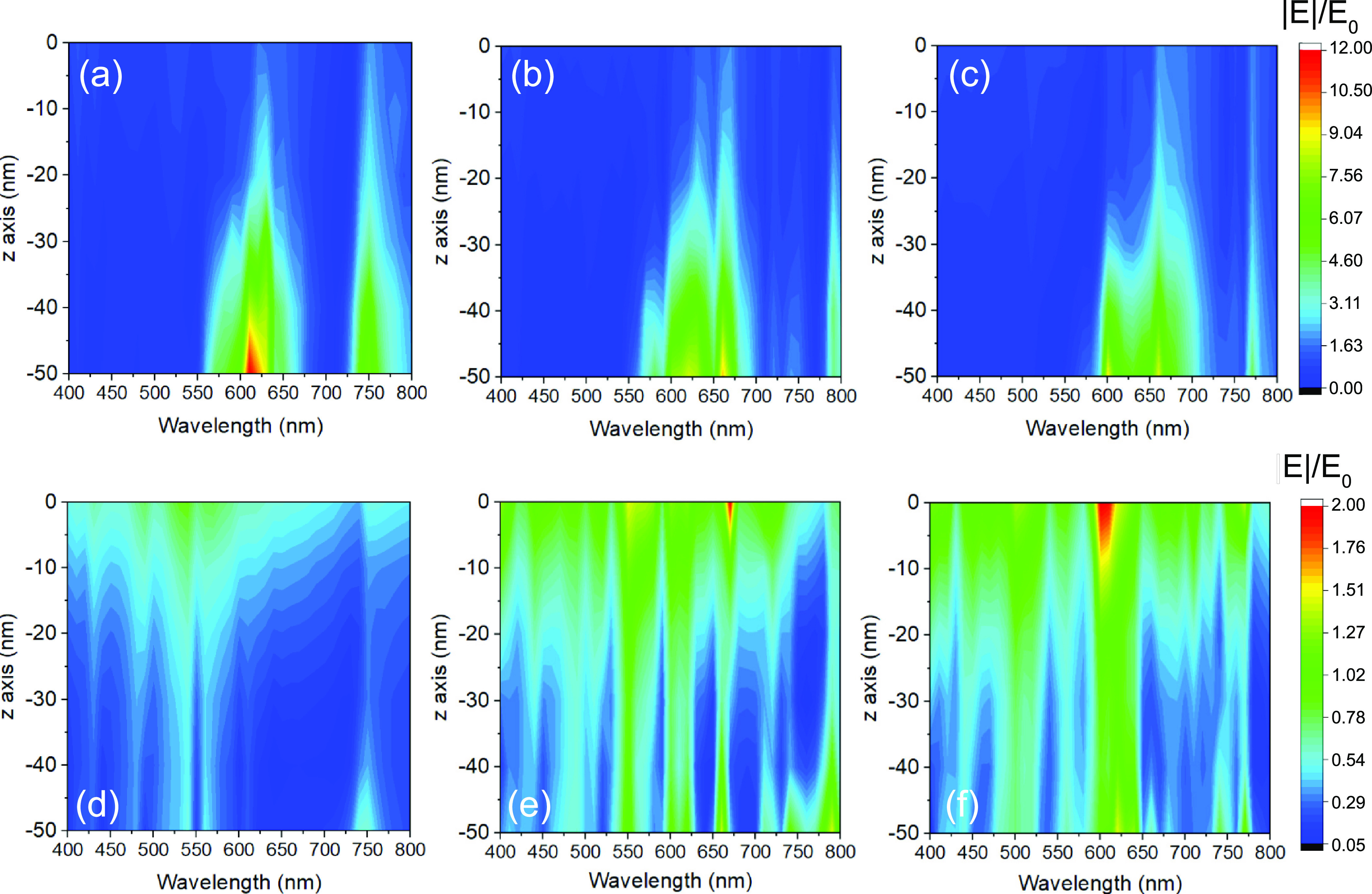
Electric field enhancement |**E**|/*E*_0_ for samples A1 (a, d), B1 (b, e), and C1 (c, f) as a function of the incident EM wave calculated in a point inside the P-NHs (top panels) and among the nanocavities (bottom panels) in the metasurface volume (0 < *z* < −50 nm, 0 nm correspond to the gold top surface).

It is well known that the field enhancement varies with the wavelength of the incident EM wave. Maps in Figure 4 obtained from a parametrized COMSOL simulation confirm this behavior for the three samples, considering two particular points on the metasurface: one located inside the P-NHs (referred to under “inside nanocavity”) and the other between the nanocavities (“gap”). The analysis reports a normalized near-field enhancement of about 12 for sample A1 (the highest obtained value), obtained in the lower vertex of the pyramidal nanohole (*z* = −50 nm) and in the spectral range 610–640 nm (Figure 4a); a second band occurs in the range 740–780 nm. In the gap area between the nanocavities, there is no significant improvement of |**E**|/*E*_0_ in the considered spectral range (Figure 4d). For sample B1, the field enhancement is more significant in the range 575–685 nm with |**E**|/*E*_0_ values around 7–9, with a second band around 785 nm (Figure 4b).

The satisfactory response of this sample over a wide spectral range in the gap region between the nanocavities (Figure 4e) is noteworthy. For sample C1, |**E**|/*E*_0_ values are in a 6–7 interval in a 580–710 nm spectral range, while a good response was found for the gap region between the nanocavities (Figure 4c,f). Through this analysis, it is possible to identify the wavelength that best couples with the structure by exploiting both the interior of the P-NHs and the gaps in-between the nanocavities.

**Figure 5:**
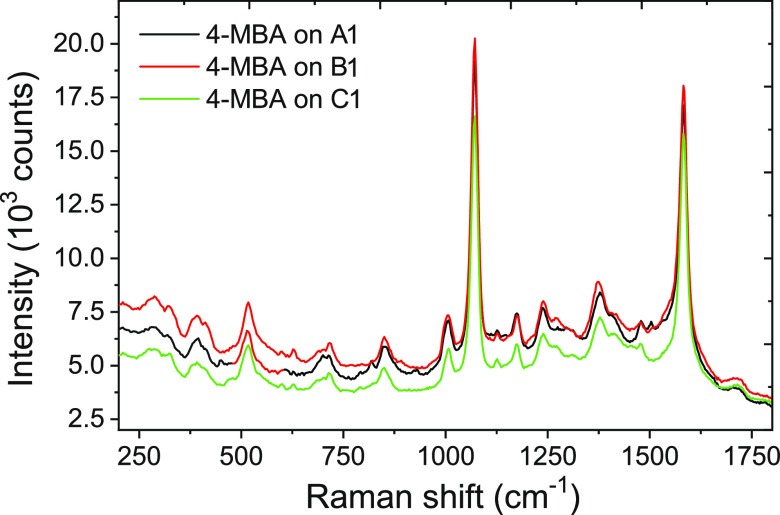
SERS spectrum of 4-mercaptobenzoic acid (4-MBA) probe on the plasmonic Au nanopyramid arrays A1, B1, and C1.

The SERS performances of the P-NHs were tested using the 785 nm excitation wavelength (Figure 5) to prove the applicability of our metasurface for different SERS analyses. For a more sensitive detection of analytes at a low concentration, the structural tunability of the surface plasmon resonances of the nanoplatforms is an essential step to allow maximization of the SERS enhancement.

For this purpose, the SERS efficiency of our nanostructures with different edge–edge interdistance was investigated using a self-assembled monolayer (SAM) of the well-known Raman probe 4-mercaptobenzoic acid (4-MBA) (see Figure 5). Furthermore, to evaluate the SERS enhancement ability of the designed plasmonic nanopatterns, the enhancement factor (EF) was calculated for the P-NHs from the spectral signal achieved of 4-MBA. EF values are shown to be 5.2 × 10^6^, 6 × 10^6^, and 4.9 × 10^6^ for samples A1, B1, and C1, respectively. It reveals that all of the fabricated P-NH substrates demonstrate a very strong SERS activity for effective Raman analysis, down to single-molecule detection. For details related to the EF calculation, see the “SERS Measurements” section and the related Supporting Information.

Figure 5 shows the SERS spectra of the Raman probe immobilized on the three nanostructures A1 (black line), B1 (red line), and C1 (green line). In all of the spectra, two main peaks of 4-MBA at 1073 and 1584 cm^–1^, associated with the CC stretching of the aromatic ring, can be clearly identified. The intensities of the three spectra fall in the same range as that of the higher value reached by nanostructure B1. This last result can be explained observing that for lower values of the edge–edge distance, the hot spot density characteristic of the nanostructures taken into account increases while the density of gold surface available for probe link decreases.

**Figure 6:**
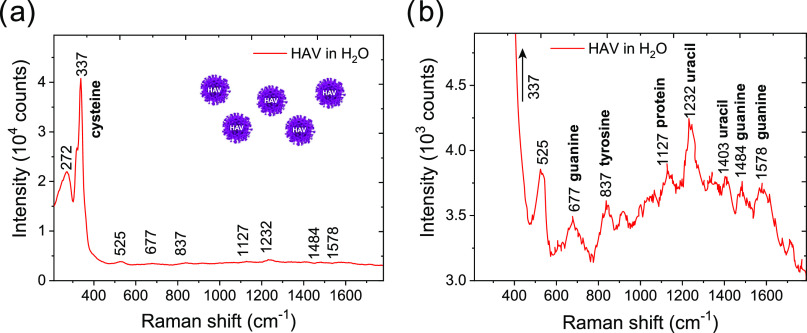
(a) SERS spectrum of the HAV virus in water (10^5^ PFU/mL) adsorbed on the gold nanocavity pattern and (b) zooming on the peaks detected between 520 and 1600 cm^–1^ with tentative assignments to chemical groups present in the analyzed sample.

As a proof of concept, we used the geometry B1 to test the suitability of our metasurface for two different SERS analyses of virus. First of all, we proved that the naked substrate can be used to obtain the HAV SERS fingerprint. Next, we tested the possibility to realize a biosensor for quantitative HAV detection upon functionalization of the metasurface with an appropriate antibody. Figure 6a further shows the SERS spectrum fingerprint of the HAV virus in 10^5^ PFU/mL adsorbed on the plasmonic P-NH pattern B1. In the full spectrum, a predominant peak at 337 cm^–1^, associable to cysteine, is well visible.

The SERS spectrum of HAV (Figure 6a,b) can be characterized by typical features of nucleic acids, amino acids, and other biological components present in viruses. The strong peak at 525 cm^–1^, for example, can be attributed to the S–S stretching mode of proteins.^55^ Spectral features of amino acids were found in the peak at 837 cm^–1^ (tyrosine).^56,57^ Spectral features of nucleic acids were found in the peaks at 677, 1484, and 1578 cm^–1^, assigned to guanine,^58,59^ and at 1232, 1403, and 1711 cm^–1^ assigned to uracil.^60^ The band at 1127 cm^–1^ (C–N and C–C stretches) is characteristic of the vibration of the proteins.^56,57^

**Figure 7:**
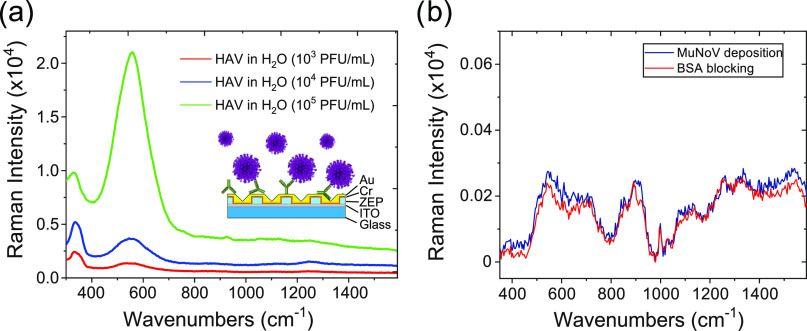
(a) SERS spectra of the HAV virus in H_2_O captured by its antibody [concentration 50 μg/mL in phosphate-buffered saline (PBS)], physically adsorbed on the nanostructure with a buffer of BSA at 3% w/w, in concentrations of plaque-forming units of the virus of 10^3^ PFU/mL (red curve), 10^4^ PFU/mL (blue curve), and 10^5^ PFU/mL (green curve). The reference peak chosen on the basis of the evident amplification is that at 550 cm^–1^. (b) Detection specificity determined after the immunosensor incubation with 10^5^ PFU/mL of a nontarget virus, the Murine Norovirus (MuNoV). The red curve refers to the spectra of the immunosensor after the BSA blocking, whereas the blue curve is acquired after the MuNoV deposition. No significant variations are appreciable between the two Raman traces.

Based on these encouraging steps, we proceeded to show that the metasurfaces studied here could also serve as SERS-based biosensors toward HAV detection. Upon physical adsorption of a monoclonal anti-HAV antibody onto the gold nanostructures and a surface passivation with 3% w/w bovine serum albumin (BSA) (see the “Virus Deposition for HAV SERS Fingerprint” section), extremely small volumes (2 μL) of HAV target were dried on the functionalized surface. After substrate washing, HAV has been detected down to a concentration of 10^3^ PFU/mL, corresponding to ≈13 pg/mL (further details are reported in the Supporting Information). Figure 7a shows the SERS spectra of the HAV in H_2_O captured by the antibody. In particular, we tested three different virus concentrations amounting to 10^3^ PFU/mL (red curve), 10^4^ PFU/mL (blue curve), and 10^5^ PFU/mL (green curve). Figure 7a presents very strong SERS signals for all increasing concentrations.

Subsequently, the peak around 550 cm^–1^ (which can be assigned to S–S stretching vibrations of disulfide bonds formed by cysteine)^56,57,61^ can be considered a reference peak to detect the presence of the virus on the plasmonic substrate taken into account.

As a preliminary result, we referenced to the zero level all of the SERS spectra and integrated the peak at 550 cm^–1^ to evaluate the intensity enhancement due to the virus binding. We then plotted the 550 cm^–1^ peak areas vs the HAV concentrations to obtain a tentative calibration curve and evaluate a rough detection limit (DL) (see the Supporting Information). However, this can only be considered as a rough estimate, while more points will be needed to provide a robust and reliable DL. The error bars were inferred from the comparison of spectra obtained at three different locations on the metasurface and on three different replicas, pointing to a satisfactory reproducibility for our system. A preliminary DL value of 5260 PFU/mL (≈68 pg/mL) was estimated from the residual standard deviation of the regression line. We believe that this result is interesting considering the literature. Indeed, to the best of our knowledge, only one work concerning the direct detection of the whole HAV virus has been published. In particular, Yang et al. described a resonance light-scattering sensor, with molecularly imprinted polymers as the recognition element, achieving a DL of 8.6 pmol/L (≈77 ng/mL).^38^

In addition, the specificity of our immunofunctionalized nanostructure has been preliminarily verified in our control experiments using 10^5^ PFU/mL MuNoV as the nontarget virus (Figure 7b).^62,63^

Contrary to the spectra recorded during the HAV incubation onto the biofunctionalized substrate, in the case of MuNoV, no enhancement of the anti-HAV spectrum is observed. The anti-HAV spectra recorded before and after the MuNoV incubation are practically identical, proving the lack of MuNoV capture by the antibody and therefore the specificity of our immunosurface (Figure 7b).

Our results open to the possibility to realize a plasmonic device for a sensitive detection of the HAV realized in a short time and in a label-free way that can be used for water analysis as a valid alternative to conventional methods.

## Materials and Methods

### Nanostructure Fabrication and Morphological Characterization

We fabricated 300 × 300 μm^2^ Au nanostructures based on periodic arrays of P-NHs by using an EBL system (Raith 150). The P-NHs are equilateral triangular based (side size *l*=390 nm) and arranged in a triangular geometry. We fabricated three patterns with the minimum interparticle distances (*d*) of 110, 160, and 250 nm. The nanostructures were fabricated realizing the conventional procedure of the EBL fabrication. A layer of an electron-sensitive resist (styrene methyl acrylate, ZEP 520 A) with a thickness of 180 nm was spin-coated on a glass substrate coated with 15 nm of indium tin oxide (ITO) and baked at 170° for 5 min. Then, it was exposed to the 13 pA electron beam current with an area dose of 27 μC/cm^2^ to generate the desired geometry designed. The patterns made of P-NHs were achieved in the resist after development in an *n*-amyl acetate solvent and then rinsed for 60 s in a 1:3 methyl isobutyl ketone:isopropyl alcohol solution (MIBK:IPA) and for 30 s in IPA. Successively, on the resist surface, 2 nm Cr and 50 nm Au films were evaporated using the SISTEC CL-400C e-beam system. Morphological characterization of the fabricated plasmonic nanostructures was performed using both scanning electron microscopy (SEM—Raith 150) and atomic force microscopy (AFM) by Bioscope Catalyst in a contactless configuration using a silicon tip (radius 16 nm).

### Near-Field Characterization: SNOM Analysis

The near-field characterization was performed with a scanning near-field optical microscope (SNOM) alpha300 Sby WITec operating in the SNOM–AFM combination mode. The transmitted signal through the three plasmonic inverted pyramidal metasurface samples has been acquired under the same experimental conditions (incident power and polarization of excitation laser source, λ_exc_ = 532 nm). The laser beam is focused on the samples through an Al-coated aperture SNOM tip characterized by an aperture of lenght 60 nm, while transmitted light is collected from the bottom by a 63× objective (NA = 0.75) and detected by a photomultiplier tube (PMT).

### Numerical Simulations: Finite Element Method

The calculation of optical properties of the inverted pyramidal metasurfaces has been performed by means of a FEM method, implemented with a commercial software (Comsol Multiphysics). For the calculation of near electric fields, an infinite array of plasmonic inverted nanopyramids have been considered. The unit cell is composed of a parallelepiped rectangle, characterized by air in the superstrate and glass in the substrate. In the middle, the metasurface is composed of a 50 nm thick Au layer in which the inverted tetrahedrons are arranged with the same orientations and distance of the samples. A TM electromagnetic wave at 532 nm propagating in the negative *z*-direction is used to excite the hot spots of the nanostructures.

### Virus Deposition for HAV SERS Fingerprint

We carried out SERS measurements of the HAV physioadsorbed on the nanostructure with *a*= 50 nm. Two microliters of HAV (HM175) in mQ H_2_O with a concentration of 10^5^ PFU/mL was dropped on the surface of the gold nanopattern at room temperature. After 2 h of incubation, the H_2_O is completely evaporated, leaving only the virus on the substrate. The substrate was washed with both phosphate-buffered saline (PBS) and deionized water to remove the unadsorbed virus and then tested with the SERS tool.

### Substrate Biofunctionalization and Virus Deposition for Immuno-SERS

We functionalized the nanopattern B1 with edge–edge distance 160 nm to realize a potentially specific SERS sensor. The functionalization process is realized using 50 μg/mL of a mouse monoclonal anti-HAV antibody (IgG3HepA(1886) from Santa Cruz Biotechnology). After 12 h of incubation, the substrates were washed as in the physioadsorbed case to remove the unfixed antibody. Subsequently, the nonspecific adsorption sites on the surface of the substrate were passivated with 10 μL of bovine serum albumin (BSA) blocking solution (3% BSA in PBS) for 1 h at room temperature, and the substrate was washed again with PBS and deionized water to remove the residual BSA. The functionalized nanopatterns were SERS tested to detect 2 μL of HAV in mQ H_2_O with different concentrations ranging from 10^3^ to 10^5^ PFU/mL. After virus deposition, the solvent was left to evaporate for ≈15 min, and then, the samples were kept in aerobic conditions for further 30 min to promote immunobonding before washing with mQ H_2_O. We repeated the virus deposition procedure on new immunofunctionalized patterns using 10^5^ PFU/mL Murine Norovirus (MuNoV, MNV-1) to test by SERS measurements the specificity of our system. In our experiments, the virus suspensions were deposited in a laboratory with biosafety level 2 (BSL-2, c/o Istituto Zooprofilattico del Mezzogiorno, Portici, Italy).

### SERS Measurements

SERS analysis of the HAV was performed using a QE Pro-Raman spectrophotometer (Ocean Optics) coupled with an upright microscope Olympus BX51 in a backscattering configuration. The system was configured for λ = 785 nm (12 mW), with a grating of 1200 lines/mm and an input slit of 50 μm. The spectra were collected in the range 200–1800 cm^–1^, with 10 s of acquisition time, a 50× (NA = 0.75) microscope objective, and a laser spot with a diameter of about 20 μm. In general, we reported an average spectrum for each tested sample. Mean spectra were calculated from at least three repeated measurements on different points of the metasurface and on three different replicas.

The enhancement factor (EF) of the plasmonic nanopatterns designed was measured evaluating the spectral signal achieved for a self-assembled monolayer (SAM) of 4-mercaptobenzoic acid (4-MBA), a molecule probe widely used in the literature to test SERS substrate. The SAM was obtained by submerging the nanostructures for 12 h in a 100 μM ethanolic solution of 4-MBA at room temperature. Subsequently, the surface was washed with water and ethanol to remove any excess of the probe molecule noncovalently bound onto gold. The EF of the nanopatterns with three different interparticle distances was evaluated as described in a previous work.^12^ In particular, for this evaluation, we used the following well-known equation: *E*_F_ = (*I*_s_ × *N*_r_)/(*I*_r_ × *N*_s_), where *I*_s_ and *I*_r_ are respectively the integrated intensities of the main SERS peaks at 1073 cm^–1^ of 4-MBA molecules adsorbed on the different substrates and 4-MBA powder, while *N*_s_ and *N*_r_ are the number of 4-MBA molecules contributing to the signal in the two cases considered at the irradiation spot of the laser, respectively.

## Conclusions

In this work, we proposed a novel plasmonic nanostructure based on high-density metallic inverted pyramidal nanohole arrays fabricated by combining a top-down process EBL and thin metallic film deposition technologies that showed remarkable sensitivity performances and enhanced electromagnetic fields. As a proof of concept of the potentialities of our nanosensor in virus detection, an extremely small volume (2 μL) of HAV target was dried on the functionalized array, and HAV has been detected at a concentration of 13 pg/mL. This new tool has the potential to provide results over short times and can be used in field or in laboratories without adequate instrumental resources for biomolecular techniques.

## Supporting Information Available

Morphological profile of a single Au nanocavity; far-field characterization: Vis–NIR spectroscopy; comparison of the optical response of the considered hexagonal arrangement P-NH metasurface and other geometries; EF calculation and SERS measurements; minimal HAV concentration detected; calibration curve and detection limit; and comparison of the pyramidal nanohole metasurface with the commercial Klarite sensors (PDF)
