# Supplementary material for: Plasmonic Metasurfaces Based on Pyramidal Nanoholes for High-Efficiency SERS Biosensing
Source: ACS Appl Mater Interfaces. 2021 Sep 1;13(36):43715–25. doi: 10.1021/acsami.1c12525 (PMC8447193; doi:10.1021/acsami.1c12525)
Supplement: Supplementary file 1 — am1c12525_si_001.pdf [file am1c12525_si_001.pdf]

Supporting Information

Plasmonic Metasurfaces based on Pyramidal Nanoholes for high efficiency SERS biosensing

Giovanna Palermo,[†,‡,#] Massimo Rippa,[¶,#] Ylli Conti,[†] Ambra Vestri,[¶] Riccardo Castagna,[¶] Giovanna Fusco,[§] Elisabetta Suffredini,[§] Jun Zhou,[‖] Joseph Zyss,[⊥] Antonio De Luca,[*,†,‡] and Lucia Petti[*,¶]

[†]Department of Physics, University of Calabria, Via P. Bucci, 87036 Rende (CS), Italy

[‡]CNR NANOTEC - Istituto di Nanotecnologia, UOS Cosenza, 87036, Rende (CS), Italy

[¶]Institute of Applied Sciences and Intelligent Systems "E. Caianiello" CNR, 80078 Pozzuoli, Italy

[§]Department of Food Safety, Nutrition and Veterinary Public Health, Istituto Superiore di Sanitá, 00161 Rome, Italy

[‖]Institute of Photonics, Faculty of Science, Ningbo University, 315211 Ningbo, People's Republic of China

[⊥] LUMIN Laboratory (CNRS), Institut d'Alembert, Université Paris Saclay, Gif sur Yvette, 91190 France

*E-mail: antonio.deluca@unical.it; l.petti@isasi.cnr.it

- Morphological profile of a single Au nanocavity
- Far-field characterization: VIS-NIR spectroscopy
- Comparison of the optical response of the considered hexagonal arrangement P-NHs metasurface and other geometries
- EF calculation and SERS measurements
- Minimal HAV concentration detected
- Calibration curve and detection limit
- Comparison of the pyramidal nanoholes metasurface with the commercial Klarite sensors

Morphological profile of a single Au nanocavity

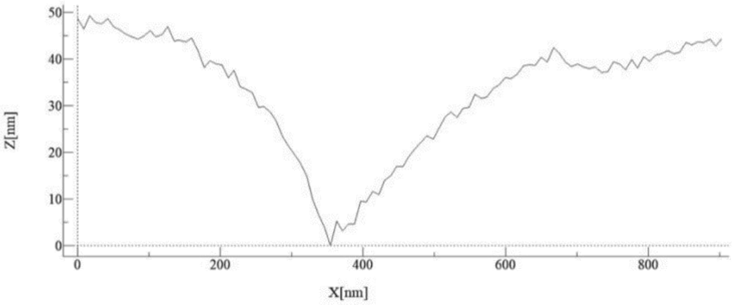

Figure S1: Morphological profile of a single Au nanocavity. The profile shows the inverted pyramidal shape, with a depth of about 50 nm.

- Far-field characterization: VIS-NIR spectroscopy

Spectroscopic characterization was performed by VIS-NIR extinction measurements that allow to investigate the characteristic Localized Surface Plasmon Resonance (LSPR) of the entire nanostructures. The used set-up was reported in a previous work [12]. The transmitted signal *T* versus wavelength λ achieved from the nanostructure is measured using as source the unpolarized white light of a halogen lamp focused on it. An 10X (N.A. = 0.25) objective was used to focus the light on the sample. The transmitted signal $T(\lambda)$ was collected by a fiber with a core of 50 μm, positioned behind the nanostructure and connected to a spectrometer (Ocean Optics USB4000, optical resolution ≈1 nm). Percentage transmission $T\%(\lambda)$ was calculated using the relation $T\%(\lambda) = (T(\lambda)-T_d) / (T_{ref}(\lambda) - T_d) \times 100$, where $T_{ref}(\lambda)$ represents a reference spectrum measured through the substrate and out of the nanostructure and T_d the dark spectrum obtained by turning off the halogen light source. Finally, extinction spectra $E_s(\lambda)$ was achieved by the expression $E_s(\lambda) = 100 - T\%(\lambda)$.

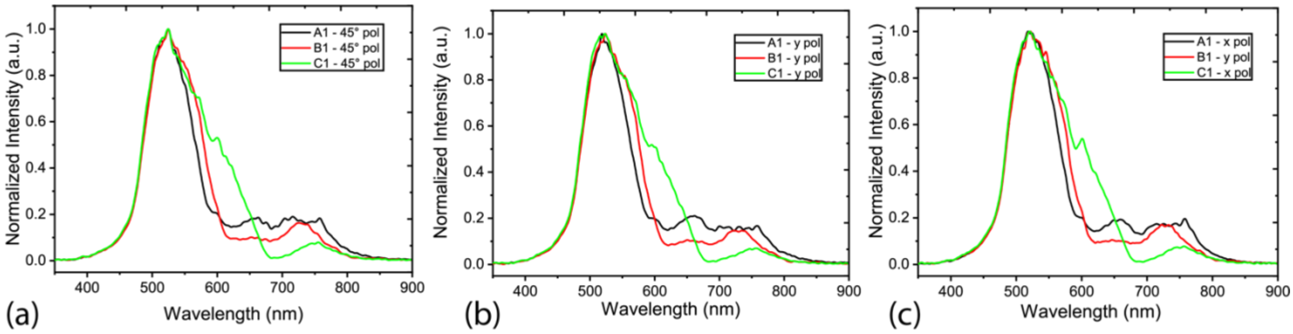

Figure S2: Extinction spectra for the samples A1, B1 and C1 for (a) 45° polarized, (b) y polarized and (c) x polarized white light.

It is possible to distinguish the typical absorbance of Au at 532 nm and a second ($\lambda_2 \approx$ 650 nm) and third ($\lambda_3 \approx$ 750 nm) plasmonic band related to the coupling between the nanocavities. By increasing the inter-distance among the nanocavities, or the lateral size, a red shift of the resonance occurs.

Supporting Information

- Comparison of the optical response of the considered hexagonal arrangement P-NHs metasurface and other geometries:

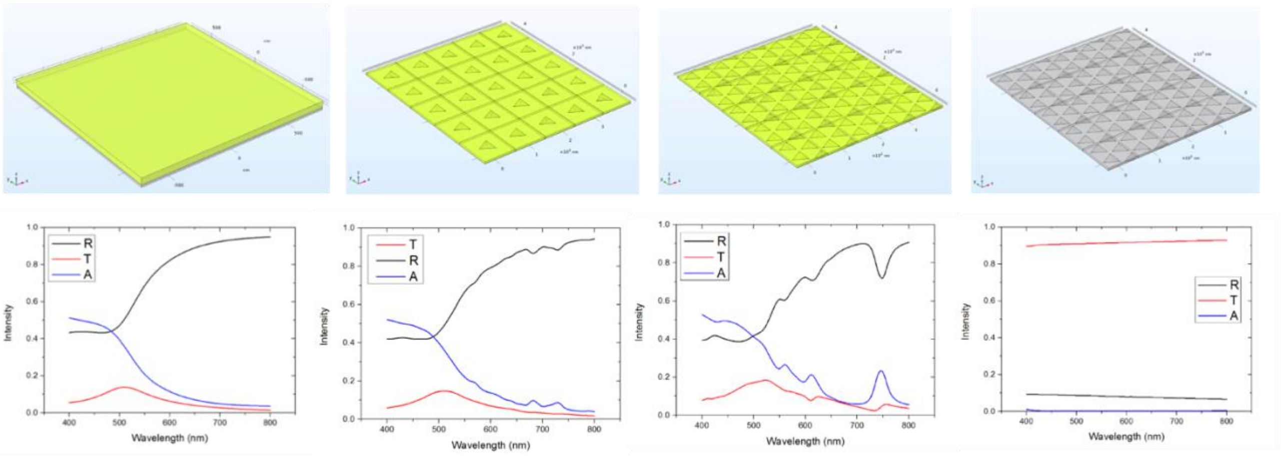

Figure S3: Transmittance (T), Absorbance (A) and Reflectance (R) for i) a slab of gold of the same thickness of the metasurface, ii) a plasmonic metasurface characterized by a different arrangement of the pyramidal nanoholes, iii) the plasmonic metasurface presented in the paper and iv) a not-plasmonic metasurface (SiO_2) characterized by the same arrangement of our structures

The hexagonal arrangement of the P-NHs in the metasurface is responsible of an optimization of the hot-spots' formation. This can be correlated to the optical response of the metasurface in terms of Reflectance, Transmittance and Absorbance. In particular, a gold slab characterized by the same thickness of our metasurface (50 nm) shows, in terms of Absorbance, the typical plasmonic band of gold in the range 400-550 nm, due to the intraband and interband transitions. If the gold slab is nanostructured, for example with P-NHs, plasmonic modes at different wavelength appear and, by changing the arrangement of the P-NHs, different coupling between the nanocavities can be obtained, with an overall increasing of the plasmonic modes intensity and a corresponding enhancement of the electric near-field. The intrinsic plasmonic nature of these modes can be demonstrated by considering the same metasurface composed by a dielectric material, in our case we consider silica (SiO_2). As we can see, the signal results to be totally transmitted and no modulation of the absorbance response is obtained in this case.

- EF calculation and SERS measurements

The Raman enhancement factor (EF) of the different SERS-active substrates was determined using the following equation:

$E_F = (I_S \times N_r)/(I_r \times N_S)$, (1)

where I_S and I_r are respectively the integrated intensities of the main SERS peak at 1073 cm^{-1} of 4MBA molecules adsorbed on the different substrates and the area of the peak at 1073 cm^{-1} of the bulk in the Raman spectrum, while N_S and N_r are the number of 4MBA molecules contributing to the signal in the two cases considered at the irradiation spot of the laser. I_r was calculated to be 2.72 x 10^3. The values of I_S were calculated to 2.81 x 10^5, 3.08 x 10^5, 2.57 x 10^5 for the three nanostructures A1, B1 and C1, respectively (SERS spectra of Fig. 4A). The number of molecules that contribute to the SERS signals is dependent on the area of the laser spot (A_{laser}), on the filling factor (FF) and on the cross section for 4-MBA (σ), as indicated in the following equation 2 [10]:

$N_s = A_{laser} * FF/\sigma$ (2)

So that the $N_s = 4.2 \times 10^6$. The number of molecules that contribute to the Raman bulk signal is given by the following equation 3 [9, 52]:

$N_r = V_{exc} * N_{Av} * D_{4MBA} / W_{4MBA}$ (3)

with the excitation volume $V_{exc} = 38.4$ µm^3, the Avogadro's number is $N_{av} = 6.022 \times 10^{23}$ mol^{-1}, the density of 4-MBA is $D_{4MBA} = 1.5$ g cm^{-3}, the molecular weight for 4-MBA is W_{4MBA} = 154,19 g mol^{-1}. So that, $N_r = 2.2 \times 10^{11}$.

Finally, the calculated value for the SERS enhancement is obtained to be 5.2x10^6, 6x10^6 and 4.9x10^6 for samples A1, B1 and C1, respectively.

- Minimal HAV concentration detected

The 10^3 PFU/mL HAV concentration was estimated to correspond to ≈ 13 pg/ml. The calculation was performed considering that 6.9 × 10^3 PFU was determined to correspond to 6 × 10^6 viral particles [58] and the viral molecular weight reported in literature for picornaviruses was 8-9 × 10^6 g/mol [59].

- Calibration curve and detection limit (DL)

The binding of the analyte on the sensor surface resulted in an increasing Raman signal. This enhancement was not exclusive for just a single peak of the spectrum, but it involved the whole spectrum. Anyway, for convenience, the authors have chosen a single peak to evaluate the signal enhancement effect due to the analyte binding to the sensor surface (the Raman enhancement of the 1077 cm^{-1} band). To evaluate the signal enhancement, all the SERS spectra are referenced to the zero level by the authors, as we did for our spectra.

Then we integrated the peak at 550 cm^{-1} to evaluate the intensity enhancement due to the virus binding. We plotted the 550 cm^{-1} peak areas vs the HAV concentrations to obtain a tentative calibration curve and evaluate a rough detection limit (DL), even though we are conscious that 3 points are not enough to construct a robust and reliable DL. The DL estimated from a residual standard deviation of the regression line was 5260 PFU/ml (≈ 68 pg/ml) and the calibration curve is reported below:

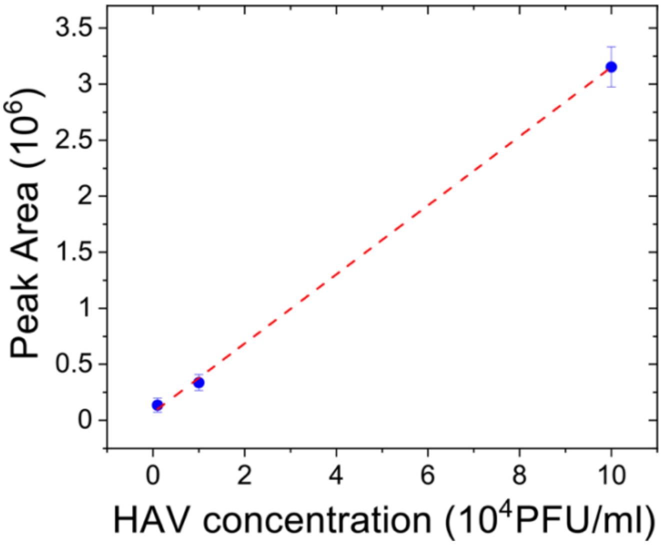

Figure S4: Calibration curve used to estimate the detection limit (DL) of our biosensor.

- Comparison of the pyramidal nanoholes metasurface with the commercial Klarite sensors

In order to better show the advantage of pyramidal nanoholes compared with commercial substrates with similar shapes, we chose to compare our structure with the Klarite sensors. Klarite sensors are well known commercial SERS substrates widely reported in literature [1-2]. Although Klarite substrates are also based on pyramidal gold arrays, there are some relevant differences compared with the P-NHs pattern proposed in this work, from both size and geometrical point of view (see Tables S1 and S2). In particular, while in the case of Klarite substrates the unit cell consists on a square-based pyramid, in the case of the proposed nanopattern the pyramid is triangular-based. This difference is important in terms of hot spot position and intensity. In fact, the vertices of the triangular base of our pyramid (characterized by an angular aperture of 60 degree) provide higher intensity hot spots than the vertices of the square base (angular aperture 90 degree) of the Klarite substrates. Moreover, the lower area of our pyramids (6.59 x 10^{-8} mm^2) compared with the Klarite substrates ones (2.16 x 10^{-6} mm^2 for the standard Klarite 302 and 2.16 x 10^{-6} mm^2 for next generation Klarite 308) favor virus detection, as reported in this study (see Table S1).

**Table S1**	**NH: base shape**	**NH: side size (nm)**	**NH: area size (mm^2)**
**Klarite 302**	square	1470 (ref.2)	2.16 x 10^{-6}
**Klarite 308**	square	454 (ref.2)	2.06 x 10^{-7}
**P-NHs pattern**	triangular	390	6.59 x 10^{-8}

Tab. 1: Shape and size relative to the NHs present in the commercial Klarite substrate (302 standard and 308 next generation) and in the P-NHs pattern proposed.

From the point of view of the periodic arrangement, while Klarite substrates are square-based, the P-NHs nanopattern that we propose is hexagonal-based. This last difference allows to have a higher packing factor of the unit cell and therefore, combined with its smaller dimensions, a higher cell's density (and therefore of hot spots) compared to the substrates of the Klarite family (see Table S2).

**Table S2**	**Cell: shape**	**Cell: side size (nm)**	**Cell: area size (mm^2)**	**Cell density (for 1 mm^2)**
**Klarite 302**	square	2040 (ref.2)	4.16 x 10^{-6}	2.4 x 10^{5}
**Klarite 308**	square	636 (ref.2)	4.04 x 10^{-7}	2.5 x 10^{6}
**P-NHs**	hexagon	275	1.96 x 10^{-7}	5.1 x 10^{6}

Tab. S2: Shape and size relative to the unit cell present in the commercial Klarite substrate (302 standard and 308 next generation) and in the P-NHs pattern proposed.

1. Analyst, 2020,145, 277-285. DOI: 10.1039/C9AN01715A
2. SPIE-Optics and Photonics West, volume 8099, paper 8099-7, San Diego, CA, 20 August 2011. "Surface Enhanced Raman Scattering (SERS)-Based Next Generation Commercially Available Substrate: Physical Characterization and Biological Application".
